# Boracic Acid in Ammoniacal Cystitis

**Published:** 1879-11

**Authors:** 


### BORACIC ACID IN AMMONIACAL CYSTITIS.

Boracic Acid has long been known to possess the property of preventing or retarding certain of the phenomena of fermentation and putrefaction. At the same time it is quite devoid of any toxic or irritating action, when absorbed into the system, or brought into contact with the tissues of the body. Hence its use in some countries for the preservation of meats for the table; hence its use in antiseptic surgery. Professor Polli, of Florence, has for several years been studying the chemical and therapeutical properties of this agent. Upwards of a drachm was administered daily, dissolved in water in the proportion of one part to fifty, without the production of any symptoms of intolerance. The drug is eliminated, unaltered, in the urine. Polli recommends its use in chronic cystitis, attended with ammoniacal decomposition of the urine within the bladder, and cites several cases in which this medication was beneficial.—*Boston Medical and Surgical Journal.*

